# The Potential of the HeartLogic^TM^ Algorithm in Patients with a Left Ventricular Assist Device, an Initial Report

**DOI:** 10.3390/jcdd11020051

**Published:** 2024-02-01

**Authors:** Michelle Feijen, Anastasia D. Egorova, Laurens F. Tops, Meindert Palmen, J. Wouter Jukema, Martin J. Schalij, Saskia L. M. A. Beeres

**Affiliations:** 1 Department of Cardiology, Leiden University Medical Center, 2333 ZA Leiden, The Netherlands; m.feijen@lumc.nl (M.F.); a.egorova@lumc.nl (A.D.E.);; 2 Department of Cardiothoracic Surgery, Leiden University Medical Center, 2333 ZA Leiden, The Netherlands; 3 Netherlands Heart Institute, 3511 EP Utrecht, The Netherlands

**Keywords:** HeartLogic^TM^, left ventricular assist device, heart failure, CIED, multisensory remote monitoring

## Abstract

Background: Survival and quality-of-life of left ventricular assist device (LVAD) recipients improved significantly because of growing experience and technological advances. However, LVAD-related complication rates, including recurrent episodes of congestion, remain high. Early detection of fluid retention to provide a time-window for medical intervention is the pillar in preventing hospitalizations. The multisensory HeartLogic^TM^ algorithm accurately detected impending congestion in ambulant heart failure patients. The aim of the current study is to investigate the feasibility of HeartLogic^TM^-driven care in LVAD patients. Methods: Consecutive LVAD destination therapy patients were followed-up according the structured HeartLogic^TM^-based heart failure carepath. An alert triggered a device check-up, and the heart failure team contacted the patient to evaluate for signs and symptoms of impending congestion. An alert was adjudicated as true positive or unexplained. An episode of congestion not preceded by an alert was deemed as a false negative. Results: Data from 7 patients were included: the median age was 67 years [IQR 61–71], 71% were male and 71% had a non-ischemic aetiology. Total follow-up entailed 12 patient-years. All patients experienced at least one alert. In total, 33 alerts were observed. Majority of alerts (70%, *n* = 23) were driven by congestion and one alerts (15%) were clinically meaningful but not primarily fluid-retention-related (e.g., altered hemodynamic triggered by a pump thrombosis). Of all the alerts, five (15%) were classified as an unexplained alert, and during follow-up, four false negative episodes were documented. Conclusions: HeartLogic^TM^-driven care with continuous monitoring to detect impending fluid retention in LVAD patients was feasible and deserves further prospective validation.

## 1. Introduction

Left ventricular assist device (LVAD) implantation is an established therapy for advanced heart failure (HF) [1]. Technological developments and growing experience improved survival and quality of life in LVAD patients [1]. Nevertheless, LVAD-related complication rates after technically successful LVAD implantation remain high [1]. One of these is recurrent congestion which can be attributable to right or left sided HF and can either have a primarily LVAD-related or non-LVAD-related cause [2]. In daily practice, right ventricular (RV) failure, aortic valve regurgitation (AR) and tricuspid valve regurgitation (TR) are the most common causes of recurrent episodes of congestion in LVAD patients and result in recurrent hospitalizations [2].

Current management of LVAD recipients is guided by symptoms, pump parameter evaluation, laboratory values and echocardiography during structured outpatient visits. Accordingly, worsening HF is frequently detected relatively late in the course. Remote cardiac monitoring is a promising approach to detect worsening HF at early stage, allowing intervention before congestion becomes symptomatic [3]. The multisensory HeartLogic^TM^ algorithm is incorporated in implantable cardiac defibrillators (ICD) and can accurately detect impending congestion in ambulatory HF patients [4,5,6,7]. Incorporating 5 ICD-based sensors into a cumulative index, HeartLogic^TM^ was able to predict episodes of congestion with a sensitivity of 70% at a median of 34 days before hospitalization in chronic HF patients [4]. As the majority of LVAD patients have an ICD, continuous ICD-based monitoring of fluid status and prediction of upcoming congestion could be promising. To date, the feasibility of HeartLogic^TM^ implementation in LVAD patients remains to be investigated. Here, in this feasibility study, we report the application of HeartLogic^TM^-driven care in LVAD patients.

## 2. Materials and Methods

The single center feasibility study included patients from January 2018 until August 2022 from the HF outpatient clinic of a university hospital. LVADs are implanted as destination therapy in this hospital. Patients were eligible for inclusion if they had a LVAD and an activated and calibrated HeartLogic^TM^ algorithm on their ICD (Boston Scientific, Marlborough, MA, USA).

Data were prospectively gathered from the patient information systems (EPD-Vision and Hix-Chipsoft, Amsterdam, The Netherlands) and the LATITUDE^TM^ platform (Boston Scientific). The baseline visit was the first outpatient clinic visit after LVAD implantation, or after calibration of the HeartLogic^TM^ algorithm.

The HeartLogic^TM^ algorithm uses five automatically collected sensors that are computed into the cumulative HeartLogic^TM^ index [4]. The algorithm includes the first and the third heart sounds (S1 and S3, respectively) and the S3/S1 ratio. The S1 sensor is a surrogate for LV-contractility and S3 a surrogate for elevated filling pressures. The third included sensor is the respiration rate and rapid shallow breathing sensor, which are markers for shortness of breath. Furthermore, thoracic impedance is as a marker for pulmonary fluid accumulation and increased night heart rate reflects the increased autonomic response of impending fluid retention. The algorithm is completed with the activity sensors which is a markers of the patients’ overall status.

**Figure 1:**
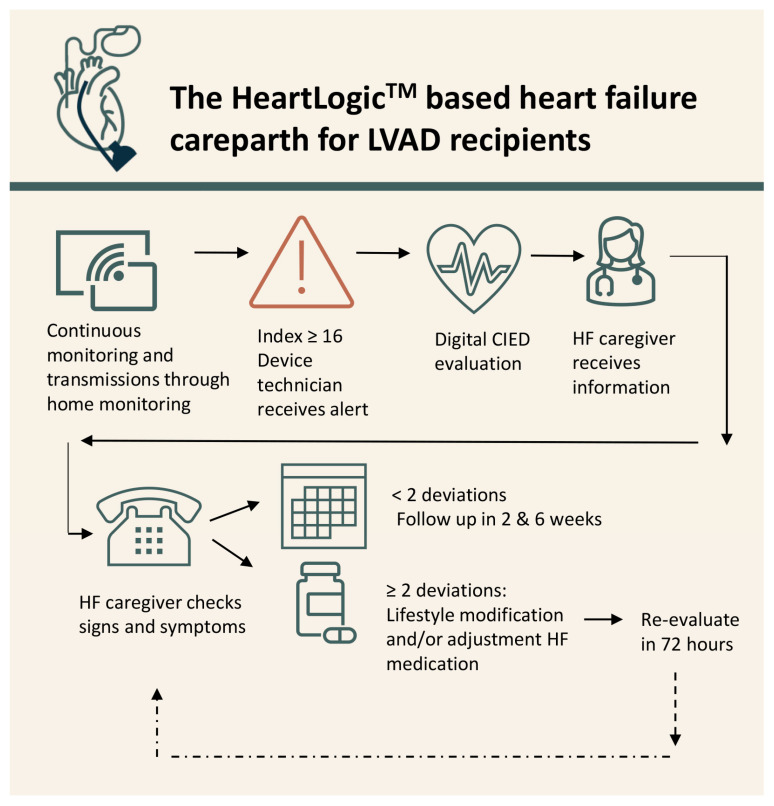
The HeartLogic^TM^-based heart failure carepath.

Patients were followed according to previously described the HeartLogic^TM^-based carepath [8]. Shortly, patients were continuously monitored via home monitoring. When the threshold of 16 was surpassed, an alert was automatically sent via LATITUDE^TM^ (Figure 1). If ICD interrogation did not detect any explanatory-device-related issues, the alert including (amongst others) arrythmia burden and biventricular pacing information was sent to the HF team. A HF nurse contacted the patient within 72 h to evaluate signs and symptoms of worsening HF and LVAD parameters.

An alert was considered true positive if ≥2 parameters suggestive of congestion (according to ESC guidelines) apart from the alert were present [9] (Table S1). Lifestyle restrictions were reinforced, and symptom severity determined further clinical action, in line with the guidelines, at discretion of the cardiologist. When medical treatment was intensified, a new digital appointment was scheduled within 72 h. In case of ≤1 suggestive parameters, the patient was scheduled for digital re-evaluation at 2 and 6 after the initial alert. If the patient developed symptoms within 6 weeks after the initial alert, treatment as previously described was initiated and the alert was deemed to be true positive. In where the index spontaneously dropped below the recovery threshold, the re-evaluations were cancelled and the alert was considered false positive. Furthermore, if ≤1 parameters of HF were documented within the 6-week follow-up window an alert was false positive. When a patient developed ≥2 parameters suggestive of congestion in the absence of an alert, the episode was regarded as a false negative.

### Ethical Statement

The study was conducted in accordance with the declaration of Helsinki, applicable local law, and the European directive for data protection (GDPR). The local scientific board approved the study and need for written informed consent was waived by the institutional medical ethical board (protocol G21.103). All patients provided consent for registration of their data and publication.

## 3. Results

In total, seven LVAD (Heartware, Medtronic, Minneapolis, MN, USA) patients with HeartLogic^TM^ were included. In three patients, HeartLogic^TM^ was activated prior to LVAD implantation, and in four, the LVAD was implanted before HeartLogic^TM^ activation. Most patients had a MOMENTUM device (*n* = 4), two patients had a RESONATE X4 and one patient had a CHARISMA X4. Total follow-up was 12 patient years (median 1.7 years [0.8–2.7]). During follow-up, no episodes of electromagnetic interference between the LVAD and the CIED were reported.

The median age was 67 years [IQR 61–71], the majority were male (*n* = 5, 71%) and most patients had a non-ischemic cardiomyopathy (*n* = 5, 71%) (Table 1). In three patients (42%), RV function was moderate–severely reduced, and three patients (29%) had a moderate–severe TR. Systolic pulmonary artery pressure (sPAP) was elevated in five patients (71%).

**Figure 2:**
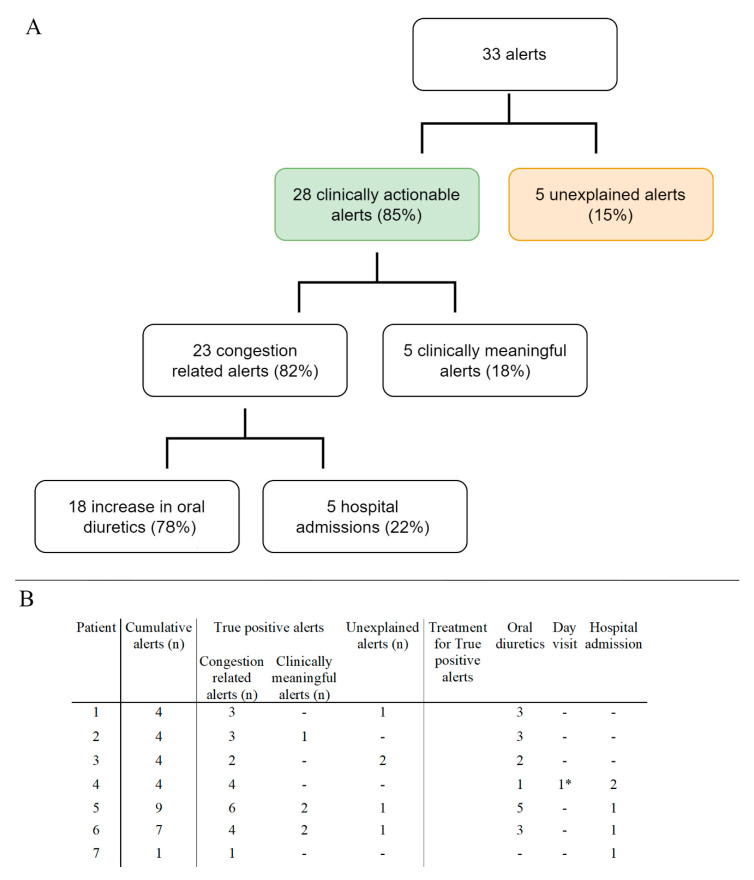
Panel (**A**). Flowchart of the number and type of alerts observed during the studied period. Panel (**B**) type and number of alerts per patient and treatment per true positive alert. * One patient required a first aid visit for an electrical cardioversion for atrial arrythmia besides an increase in oral diuretics.

During follow-up, 33 alerts occurred (Figure 2A). All patients experienced at least 1 alert, 4 (57%) experienced 4 alerts and 2 (29%) had >4 alerts, leading to 2.7 alerts/patient year (PPY). The median alert duration was 39 days [IQR 27–69]. Of all alerts, 85% (*n* = 28) were clinically actionable. Majority (*n* = 23, 70%) of alerts were congestion related (Figure 2B). The most common causes of worsening HF were excessive fluid and/or salt intake, ventricular tachycardia, and progressive RV failure. An increase in oral diuretic treatment, in adjunction with lifestyle restrictions reinforcement, was sufficient for most alerts (*n* = 17, 74%). On top of that, one (4%) patient needed an electrical cardioversion to restore sinus rhythm and recompensate. In five (22%) alerts, hospitalization for intravenous diuretic treatment was required, despite an initial increase in oral diuretic treatment. In those hospitalized, the median duration of hospitalization was 3 days. Furthermore, five (15%) alerts were clinically meaningful, but not primary congestion related (e.g., triggered by pump thrombosis or anaemia). Of all the alerts, five (15%) were classified as unexplained alerts (UAR). The UAR was 0.42 PPY. During the follow-up of 12 patient-years, four episodes of congestion without a HeartLogic^TM^ alert were observed; these were treated with an increase in oral medication. The false negative rate was 0.33 PPY.

### Case Presentation

**Figure 3:**
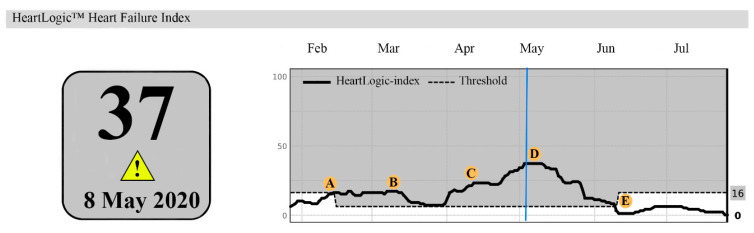
A HeartLogic^TM^ report. A: (14 February 2020) The index surpassed the threshold. B: (28 February 2020), HeartLogic^TM^ index remained stable. C: (12 April 2020): the index increased to 23. D: (3 May 2020) the index further increased to 37. E: (7 June 2020) gradual decline of the HeartLogic^TM^ index to 0. The blue line highlights the maximum value of the HeartLogic^TM^ index.

In 2006, a 58-year-old female was diagnosed with a non-ischemic cardiomyopathy. A CRT-D was implanted in 2008 and a destination therapy LVAD in 2019. On outpatient visit (2020), echocardiography showed mild AR, moderately reduced RV function, moderate–severe TR and estimated sPAP 44mmHg. Serum NT-Pro BNP was 2805 ng/L and creatinine 177 μmol/L. The HeartLogic^TM^ index surpassed the alert threshold on 14 February 2020. Then, she denied symptoms of congestion (Figure 3). She remained asymptomatic at 2 weeks follow-up (B). At 4 weeks follow-up the index increased to 23 and she reported weight gain and dyspnoea (C). Bumetanide dosage was doubled. Symptoms improved and target weight was reached within a week. The HeartLogic^TM^ index declined to 17 and bumetanide was decreased. Two weeks later, the index raised to 37 and she reported dyspnoea and oedema (D). Bumetanide dosage was escalated, and the index gradually declined to below the threshold (E).

## 4. Discussion

This study evaluates HeartLogic^TM^-driven HF care in LVAD patients. The main finding is that HeartLogic^TM^ has the potential to detect impending fluid retention in LVAD patients, with a low UAR. Furthermore, the incidence of false negative alerts is low. These results support the concept of continuous-device-based multisensory monitoring in LVAD patients to facilitate early detection and adequate treatment of congestion.

In patients with advanced HF, LVAD therapy provides survival benefit and improves quality of life [1]. However, congestion-related (re-)admission rates remain high [2]. Strategies to timely identify and intervene in patients at risk for recurrent congestion are therefore warranted. One of the possible strategies is remote cardiac monitoring, since most LVAD patients have an ICD [9]. This concept is studied in a brief report previously, but did not provide substantial evidence for direct implementation [10].

The unique anatomical and physiological aspects of LVAD patients make the efficacy of HeartLogic^TM^ in these patients particularly interesting. As mentioned, the multisensory HeartLogic^TM^ index is based on accelerometer-based heart sounds [4]. The use of these measurements is validated in HF patients without an LVAD [11]. Accurate analysis of heart sounds may be hampered by the mechanical LVAD humming. However, Chen et al. previously demonstrated that accelerometer-based detection of heart sounds in LVAD patients is feasible [12]. In the current study, six out of seven patients had continuous heart sound data available. In one case, S3 was sporadically unavailable. RV failure and progressive TR are frequent causes of fluid overload in LVAD patients. It could be speculated that right sided heart failure may also decrease thoracic impedance and increase respiratory rate (e.g., by pleural effusion), but this remains to be investigated in the clinical setting. This highlights the importance of the multisensory nature of the algorithm and calls for further evaluation of individual sensor contribution. In the night heart rate and activity sensors no specific LVAD related issues are expected or observed in the current analysis.

HeartLogic^TM^ was able to adequately detect impending congestion in LVAD patients when implemented in a HF carepath. Of all the alerts, 85% was clinically meaningful and triggered clinical action. Furthermore, few patients were admitted because of worsening heart failure. On top of that, the overall duration of the hospital admission was short (median 3 days), suggesting that HeartLogic^TM^-driven care facilitates quicker recompensation. Another explanation for this phenomenon is that these patients are strictly followed and admitted at an earlier stage of decompensation. Timely clinical action is empowered by early notifications of clinical deterioration.

Alerts that were not primarily caused by HF are important since relevant medical issues were detected early and enabled clinical action. Another important finding is the low incidence of false negative episodes. Accordingly, patients that are not in alert status are correctly identified by the algorithm as having a low risk for impending congestion.

Since LVAD patients travel distance to the nearest specialized hospital can be far away, remote monitoring combined with a digital consult may replace in office visits in the future in patients that are not in alert. This could contribute to a more streamlined follow-up approach and might be less disruptive for the patient’s life. On the other hand, LVAD patients frequently visit the heart failure outpatient clinic and/or other outpatient clinics. Thus, it could be argued that the benefit of remote monitoring could be less when compared to ambulant heart failure patients.

Furthermore, it could be speculated that multisensory CIED-guided heart failure care potentially shortens or avoid heart failure events, leading to improved quality of life. Finally, the feeling of being continuously monitored might alleviate concerns regarding a future heart failure episode. Current findings justify prospective validation in larger cohort of LVAD patients.

### 4.1. Limitations

Several limitations should be considered when interpreting the results of the current study. The study reports single-center observational data on the feasibility of HeartLogic^TM^ triggered carepath in LVAD patients, without a control group. The number of patients included is modest; however, the follow-up does entail 12 patient years and a reasonable number of alerts and clinical events. Although the results of the current study are only exploratory, the findings justify further prospective evaluation in larger patient cohorts, preferably in a randomized setting. The tertiary hospital in which the patients were under follow-up has extensive experience in eHealth ad remote monitoring as well as structured alert triggered carepaths; therefore, it remains to be seen whether the results will be reproducible in different hospital and healthcare systems. The HeartLogic algorithm is available on selected Boston Scientific devices, future investigations are also likely to include multisensory heart failure algorithms from other ICD brands.

### 4.2. Future Perspectives

This study demonstrated that HeartLogic^TM^-based monitoring is feasible in LVAD patients. Further analysis to investigate the effect of HeartLogic^TM^-based monitoring on hospitalizations and mortality, preferably in a randomized controlled setting, is warranted in this complex population. On top of that, it remains to be investigated whether HeartLogic^TM^ is cost-effective. This brief communication, therefore, is a call for further prospective analysis. Following the implementation of HeartLogic^TM^, multiparametric algorithms from other vendors (Triage-HF, Medtronic, Minneapolis, MN, USA and HeartInsight, Biotronik, Berlin, Germany) have been developed, which potentially enables a broader group of LVAD patients with a CIED to benefit. Finally, it remains to be investigated whether the current results are generalizable to HeartMate III recipients.

## 5. Conclusions

Device-based continuous multisensory monitoring with HeartLogic^TM^ to detect impending fluid retention is feasible and a promising tool in LVAD patients. Early detection of congestion enables prompt clinical action and may reduce the number of hospitalizations in these patients.

## Figures and Tables

**Table 1:** Baseline characteristics.

Patient	Age Inclusion (y)	Age at LVAD Implant (y)	Sex	Aetiology HF	Rhythm	NT-Pro BNP (ng/L)	Hb (mmol/L)	Creatinine (Umol/L)	LVAD Speed (rpm)	LVAD Power (watt)	LVAD Flow (L/min)	BB	ACE/ARB/ARNI	MRA	Loop Diuretics	RVF	Tapse (mm)	RVEDd (mm)	TR	sPAP (mmHg)	AR
1	61	61	F	Non-ischemic	SR	4143	8.0	123	2500	3.4	3.5	Yes	Yes	Yes	Yes	Moderately reduced	14	43	none	25	none
2	56	56	M	Ischemic	SR	1986	8.2	125	2400	3.2	3.9	Yes	Yes	Yes	Yes	Mildly reduced	16	41	none	25	none
3	65	64	M	Ischemic	SR	1184	7.9	105	2300	2.7	3.1	Yes	Yes	Yes	Yes	Mildly reduced	-	43	none	30	none
4	71	70	M	Non-ischemic	AF	7939	8.7	200	2400	3.1	3.3	No	No	Yes	Yes	Moderately reduced	-	53	none	45	none
5	72	72	F	Non-ischemic	AP	4709	6.8	181	2600	3	2.2	Yes	No	Yes	Yes	Moderately reduced	12	50	severe	30	mild
6	67	63	M	Non-ischemic	SR	1520	6.7	172	2400	2.9	2.9	Yes	No	Yes	Yes	Mildly reduced	-	52	mild	50	moderate
7	67	64	M	Non-ischemic	AP	946	4.3	126	2500	3.2	3.1	No	No	Yes	Yes	Mildly reduced	12	64	moderate	40	none

## Data Availability

The data presented in this study are available on request from the corresponding author. The data are not publicly available due to privacy restrictions.

## Supplementary Materials

The following supporting information can be downloaded at: https://www.mdpi.com/article/10.3390/jcdd11020051/s1, Table S1: Parameters suggestive of congestion.
