# Supplementary material for: The Potential of the HeartLogicTM Algorithm in Patients with a Left Ventricular Assist Device, an Initial Report
Source: J Cardiovasc Dev Dis. 2024 Feb 1;11(2):51. doi: 10.3390/jcdd11020051 (PMC10889511; doi:10.3390/jcdd11020051)
Supplement: Supplementary file 1 [file jcdd-11-00051-s001.zip › jcdd-2825089-supplementary.pdf]

Supplementary table S1.

Parameters suggestive of congestion

Parameters suggestive of congestion	
Shortness of breath last night	
Shortness of breath during the past 24 hours	
Edema in the legs or abdomen	
Shortness of breath when lying down	
Feeling unwell during the past 24 hours	
Increased liquid oral intake during the past 24 hours	
Excessive salt intake during the past 24 hours	
Reduced exercise intolerance	
Fatigue or tiredness during the past 24 hours	
Nocturnal cough	
Decreased intake during past 24 hours/ loss of appetite	
Dizziness or lightheadedness	
